# 814 Devices measuring transepidermal water loss of the skin: a systematic review of measurement properties

**DOI:** 10.1093/jbcr/irac012.362

**Published:** 2022-03-23

**Authors:** Tanja Klotz, Abdullah Ibrahim, Guy Maddern, Yugesh Caplash, Marcus Wagstaff

**Affiliations:** Royal Adelaide Hospital, Panorama, Adelaide, South Australia; Royal Adelaide Hospital, Adelaide, South Australia; University of Adelaide, Woodville, South Australia; Royal Adelaide Hospital, Adelaide, South Australia; Royal Adelaide Hospital, Adelaide, South Australia

## Abstract

**Introduction:**

TEWL is a physiological property of skin which increases when the epidermis is damaged. It is, therefore, a commonly utilised measure of skin barrier integrity. Devices measuring TEWL are available as open, semi-open or closed chamber. Studies of reliability examine the consistency of measurement, and/or responsive whereas measurement error scores in absolute terms the amount of error due to sources of variation.

**Methods:**

The search strategy aimed to locate published and unpublished studies. Databases searched included PubMed, Embase, CINAHL and Web of Science, utilising identified keywords and limited to studies in English. Grey literature sources were searched to identify any unpublished documents. Study selection using the inclusion criteria was then assessed by two reviewers for methodological quality utilising the COSMIN risk of bias tool to assess the reliability and measurement error of outcome measurement instruments.

Studies examining the reliability and/or measurement error of TEWL measurement devices were included. Studies that only report on measurement of TEWL outcomes without examination of reliability and/or measurement error were excluded.

**Results:**

A total of 22 devices were examined in the 38 included studies. The quality of study design was on average rated as ‘Adequate’ however reliability and measurement error statistical methods were on average rated as ‘Doubtful’.

**Conclusions:**

TEWL measurement devices were found to demonstrate good reliability and often correlated with other devices. However, measurement error was highly variable but improves under *in-vitro* conditions.

